# Effect of New Zealand Blackcurrant Extract on Repeated Cycling Time Trial Performance

**DOI:** 10.3390/sports5020025

**Published:** 2017-05-09

**Authors:** Connor A. Murphy, Matthew D. Cook, Mark E.T. Willems

**Affiliations:** 1 Department of Sport and Exercise Sciences, University of Chichester, College Lane, Chichester, West Sussex PO19 6PE, UK; cmurphy5@stu.chi.ac.uk; 2 Institute of Sport and Exercise Science, University of Worcester, Henwick Grove, Worcester WR2 6AJ, UK; matthew.cook@worc.ac.uk

**Keywords:** sport nutrition, anthocyanins, polyphenols, cycling performance, fatigue

## Abstract

New Zealand blackcurrant (NZBC) extract increased 16.1 km cycling time trial performance. The aim of the present study was to examine the effect of NZBC extract on 2 × 4 km time trial performance. Ten male cyclists (age: 30 ± 12 years, body mass: 74 ± 9 kg, height: 179 ± 7 cm, body fat: 11 ± 3%, $\dot{V}$O_2max_: 55 ± 7 mL·kg^−1^·min^−1^, mean ± SD) volunteered. Participants were familiarized with the time trials. Participants consumed capsulated NZBC extract (300 mg·day^−1^ CurraNZ™; containing 105 mg anthocyanin) or placebo for seven days (double blind, randomised, cross-over design, wash-out at least seven days) before 2 × 4 km time trials (10 min active self-paced recovery between trials) (SRM ergometer, SRM International, Germany). Heart rate was recorded and blood lactate sampled immediately after each trial and 8 min into recovery between the trials. Times over comparable one km distances in each 4 km time trial were similar. No effect was observed for the time to complete the first (placebo: 380 ± 28 s, NZBC: 377 ± 27 s) and second 4 km of cycling (placebo: 391 ± 32 s, NZBC: 387 ± 30 s), within both groups the second 4 km times slower by 11 ± 8 s and 11 ± 9 s for placebo and NZBC, respectively. However, the total time of the two 4 km cycling trials was 0.82% faster with NZBC extract (placebo: 771 ± 60 s, NZBC 764 ± 56 s, *p* = 0.034) with seven participants having faster total times. There was no effect of NZBC on heart rate and lactate values at identical time points. New Zealand blackcurrant extract seems to be beneficial in repeated short-distance cycling time trials for overall performance.

## 1. Introduction

Anthocyanins are water-soluble polyphenols with anti-inflammatory [1] and anti-oxidant properties [2]. Blackcurrant is high in content of the anthocyanins cyanidin-3-glucoside, cyanidin-3-rutinoside, delphinidin-3-glucoside, and delphinidin-3-rutinoside. It was shown that blackcurrant intake increased forearm blood flow following venous occlusion and reduces muscle stiffness and fatigue from typing work [3]. In addition, blackcurrant nectar intake reduced muscle damage and inflammation from eccentric knee extensions [4]. The anti-oxidant capacity of the blackcurrant anthocyanins may reduce oxidative stress during exercise [5], counteracting potential oxidative stress-related fatigue mechanisms [6]. In general, reductions in oxidative stress may improve performance due to lower depressed activity of the sodium–potassium pump activity by reactive oxygen species [7]. Therefore, the potential ergogenic effect of blackcurrant intake on physiological responses during exercise and performance is warranted. Recently, we observed that the intake of New Zealand blackcurrant powder shifted the lactate curve downward and rightward in endurance trained cyclists [8], an observation that is common following endurance training. In addition, intake of a New Zealand blackcurrant extract enhanced fat oxidation and improved 16.1 km time trial performance [9]. Besides the ergogenic effect on endurance performance, subsequent studies have shown improved performance in high-intensity treadmill running [10] and ability to reduce slowing of maximal sprint speed in the Loughborough intermittent shuttle test, but only in the latter stages of the test [11]. The effects of New Zealand blackcurrant on repeated high-intensity exercise tasks have not been examined.

Previous observations on the ergogenic effect of anthocyanins on exercise performance [9,10,11] support the hypothesis that intake of New Zealand blackcurrant extract will result in faster performance times. The aim of the present study was to examine the effects of short term intake (seven days) of New Zealand blackcurrant extract on two successive 4 km cycling time trials.

## 2. Methods

Ten male trained cyclists (Table 1) participated in the study. Participants had more than three years of cycling experience as members of local cycling clubs or teams and were not taking any other supplements. Informed consent was provided and ethical approval was obtained from the University of Chichester Ethics Committee (Code: 1516_13).

### 2.1. Experimental Design

Participants attended three testing sessions at approximately similar times during the day. Exercise testing was performed on an SRM ergometer (Schoberer Rad Meßtechnik, Jülich, Germany). Participants used their own cycling shoes and pedals, with the saddle height, saddle setback, handlebar reach, and drop set up to individual preferences and kept identical for all testing sessions. During the first session, the following participant characteristics were collected; height (Seca 213, Seca, Birmingham, UK), body mass (Kern ITB, Kern, Germany), and body fat percentage (Tanita BC418 Segmental Body Composition Analyser, Tanita, IL, USA). Subsequently, an incremental cycling test to exhaustion was completed to obtain maximal oxygen uptake. This was followed by a familiarization of the 20 min warm up protocol and two successive 4 km time trials. The warm up protocol is recommended by the British Cycling Federation for indoor sessions. The warm-ups were completed in gear three and involved various stages, where participants maintained a specific cadence. This study used a cross-over design—i.e., the same participants were tested with New Zealand blackcurrant intake and placebo. Before sessions two and three, participants consumed one capsule of either a New Zealand blackcurrant supplement (300 mg·day^−1^ CurraNZ containing 105 mg anthocyanin) or a placebo (300 mg microcrystalline cellulose M102) per day, for seven days. Participants were advised to take the capsule at the same time of the day with the last capsule taken two hours before testing. A wash out period of seven days was used.

### 2.2. Physical Activity and Dietary Standardisation

Participants completed a food diary for two days prior to testing sessions two and three. Nutritional intake by the participants was not controlled, but the food diary before session two was used by the participants to guide replication of food intake for the third session. Food diaries were analysed using Nutritics (Nutritics Ltd., Dublin, Ireland) for macronutrient intake with carbohydrate, fat, and protein intake for the two days before testing being 524 ± 139, 154 ± 70, and 241 ± 98 g , respectively (*N* = 8). Participants also completed a food frequency questionnaire with focus on fruit and vegetable intake to estimate daily normal anthocyanin intake using Phenol Explorer (http://phenol-explorer.eu/) [12]. Daily anthocyanin intake was calculated as the sum of consumption frequency of each food multiplied by the anthocyanin for the portion size and estimated to be 15 ± 12 mg·day^−1^ (*N* = 9). Before all testing sessions, participants were instructed not to perform intense exercise for the 48 h before testing, no alcohol intake 24 h before testing and no caffeine intake on the day of testing.

### 2.3. Maximal Oxygen Uptake ($\dot{V}$O_2max_) Protocol

The incremental cycling protocol for measurement of $\dot{V}$O_2max_ started with 50 W for four minutes with 30 W·min^−1^ increases until volitional exhaustion. During the protocol, participants were required to maintain a constant cadence between 70 and 90 rpm. Expired air was collected with the Douglas bag method for at least the last three minutes of the test in separate bags. Douglas bag contents were analysed with a three-point calibrated analyser (Servomex, series 1400, Crowborough, UK) for fractions of oxygen and carbon dioxide and volume with a dry gas meter (Harvard Aparatus Ltd., Edenbridge, UK).

### 2.4. Time Trials

Participants completed two 4 km time trials (gear three or four, standardized between sessions), separated by 10 min of active recovery at self-selected cycling intensity. For the time trials, the SRM ergometer was fitted with the large flywheel to simulate road cycling. During the time trials, participants had distance feedback only and were provided with standardized verbal encouragement. Finger prick blood samples were taken immediately after each time trial and eight min into recovery and analysed for plasma lactate (YSI 2300 Stat Plus, Yellow Springs Instruments Co. Inc., Yellow Springs, OH, USA). SRM software was used for data collection and provided time to completion, kilometre times (1, 2, 3 and 4) and heart rate data. Repeated 4 km time trials have no ecological validity but were performed to examine also the ergogenic effect of New Zealand blackcurrant when initiating a 4 km in a fatigued state.

### 2.5. Data Analysis

All data was analysed with SPSS 20.0 (SPSS, Chicago, IL, USA). A repeated measures two-way ANOVA was used for blood lactate, heart rate, and kilometre values in the first and second time trial for time and power with post-hoc two-tailed Student *t*-test. Total time trial times were analyzed with one-tailed Student *t*-tests. Statistical significance was accepted at *p* < 0.05. Interpretation of 0.05 > *p* ≤ 0.1 was according to guidelines by Curran-Everett & Benos [13]. Data are presented as mean ± SD unless stated otherwise.

## 3. Results

### 3.1. Cycling Performance

**Figure 1:**
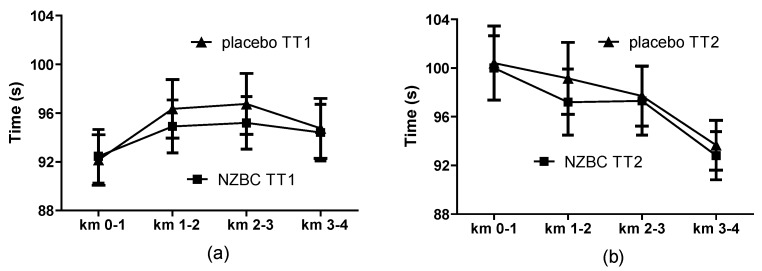
Kilometre times during the first 4 km (TT1) (**a**) and second 4 km time trial (TT2) (**b**) NZBC, New Zealand blackcurrant extract.

The total time of the two 4 km cycling trials was 0.82% faster with NZBC extract (placebo: 771 ± 60 s, NZBC 764 ± 56 s, *p* = 0.034) with seven participants having faster total times. There were no differences for the times over comparable one kilometer distances in the first (Figure 1a) and second 4 km (Figure 1b) time trial, suggesting no effect of NZBC extract on pacing strategy during the first and second 4 km time trial. No effect of NZBC extract was observed for the time to complete the first (placebo: 380 ± 28 s, NZBC: 377 ± 27 s) and second 4 km of cycling (placebo: 391 ± 32 s, NZBC: 387 ± 30 s). Compared to the first 4 km time trial, slower times were observed for the second 4 km of cycling by 11 ± 8 s and 11 ± 9 s for placebo (*p* = 0.003) and NZBC (*p* = 0.008), respectively. There was a trend for mean power over the two 4 km cycling trials to be 7 Watts higher with NZBC extract (placebo: 747 ± 138 W, NZBC 754 ± 140 W, *p* = 0.095) with seven participants having higher mean power. There was no effect of NZBC extract on the mean power for completion of the first (placebo: 390 ± 66 W, NZBC: 390 ± 69 W) and second 4 km of cycling (placebo: 357 ± 72 W, NZBC 365 ± 71 W), with both placebo and NZBC testing lower with mean power values for the second 4 km of cycling.

### 3.2. Lactate and Heart Rate

There was no effect of NZBC extract on lactate after the first 4 km time trial (placebo: 8.7 ± 1.9 mmol·L^−1^, NZBC: 9.1 ± 1.7 mmol·L^−1^, *N* = 8), 8 min during recovery between the time trials (placebo: 6.4 ± 2.5 mmol·L^−1^, NZBC: 6.2 ± 1.5 mmol·L^−1^, *N* = 8), and after the second 4 km time trial (placebo: 8.1 ± 3.1 mmol·L^−1^, NZBC: 8.5 ± 2.7 mmol·L^−1^, *N* = 8). There was no effect of NZBC extract on heart rate during the first 4 km time trial (placebo: 175 ± 13 beats·min^−1^, NZBC: 175 ± 11 beats·min^−1^, *N* = 9) and second 4 km time trial (placebo: 170 ± 10 beats·min^−1^, NZBC: 170 ± 10 beats·min^−1^, *N* = 9).

## 4. Discussion

The main finding of the present study was that New Zealand blackcurrant extract reduced the total time to complete two successive 4 km cycling time trials with an active recovery of 10 min between the time trials. In a previous study [11], the ergogenic effects on performance by New Zealand blackcurrant were observed in a more fatigued state. In the present study, total time of the two successive 4 km cycling time trials was 0.82% faster by the intake of New Zealand blackcurrant extract, with 7 out of 10 participants having faster overall times. It is likely that the second 4 km time trial was performed in a more fatigued state. Lactate responses after the cycling time trials and during the active recovery, and heart rate during the first and second 4 km cycling time trial were not affected by the intake of New Zealand blackcurrant extract.

Reductions in exercise-induced oxidative stress by the intake of New Zealand blackcurrant may explain the overall faster 4 km cycling time. Previous work by Lyall et al. [5] showed that New Zealand blackcurrant extract (anthocyanin content ~240 mg) reduced evidence of a marker of oxidation stress, i.e., lower plasma protein carbonyl levels were observed following a 30-min row at 80% $\dot{V}$O_2max_. It should be noted, however, that participants in the present study were supplemented with ~105 mg of anthocyanins for seven days before being tested. In addition, we do not know whether New Zealand blackcurrant extract under the conditions of the present study would reduce potential markers of exercise-induced oxidative stress. The presence of exercise-induced oxidative stress may affect the sodium-potassium pump activity and contribute to skeletal muscle fatigue [7]. McKenna et al. [7] observed that *N*-acetylcysteine—an anti-oxidant compound—reduced the decline in the maximal activity of the sodium-potassium pump. It would be of interest to examine the effect of New Zealand blackcurrant intake on activity of the sodium-potassium pump as a mechanism to enhance high-intensity performance. The individual responses to the potential anti-oxidant effects of New Zealand blackcurrant during high-intensity cycling may be due to individual oxidative stress responses [14]. The oxidative stress response from an isokinetic eccentric exercise with the knee extensors showed substantial individual variability [14] and potential effects of New Zealand blackcurrant may only be expected for those individuals experiencing exercise-induced oxidative stress from the successive 4 km cycling time trials.

In a study by Lansley et al. [15] on the effects of beetroot juice on 4 km cycling time trial performance in nine male cyclists, the performance time for the placebo condition was 387 ± 25 s and similar to the present study (i.e., 380 ± 28 s). However, Lansley et al. [15] observed a 2.8% improvement with a time of 376 ± 21 s, an improvement by 10 seconds. In the study by Lansley et al. [15], at least two familiarization trials were completed with the time trials repeated until the difference in completion time was less than 1%, probably resulting in reduced variation compared to the present study in which only one familiarization trial was completed. The importance of familiarization was also observed for well-trained rowers in which the third 2000 m rowing performance was still improved [16]. In that study, the time of the second row of 2000 m was 6 min and 54 s and was still improved by 0.9% (i.e., 3 s) in the third row. We cannot exclude that more familiarization should have been implemented in the present study. However, it remains also possible that New Zealand blackcurrant is just less ergogenic than beetroot juice for 4 km cycling time trial performance.

Intake of pomegranate enhanced blood flow in the brachial artery and improved performance for a treadmill run to exhaustion at 90% of peak velocity, an exercise modality with a performance time of 346 ± 162.5 s in 19 (9 female) highly active participants [17]. The performance time of this exercise modality resembles the performance time of the 4 km cycling time trial. Intake of blackcurrant has been shown also to enhance blood flow in the forearm following venous occlusion [3]. Therefore, it is possible in the present study that enhanced blood flow by New Zealand blackcurrant extract during the cycling but also in the recovery phase between repeated high-intensity cycling bouts may have somewhat enhanced the overall performance time of repeated 4 km time trials. Enhanced blood flow during the cycling may reduce the contribution of anaerobic energy production with reduced production of metabolic by-products causing fatigue.

Future studies may want to examine the effects of different polyphenol supplements on physiological responses and exercise performance. Mechanisms for the performance effects by polyphenol intake are complex due to the availability of many different metabolites in the blood [18] with potential for affecting cell function [19] and enhancing performance. Enhanced performance by polyphenol intake (for reviews see [20,21]) is likely explained by physiological effects specific for the type, amount, and duration of polyphenol intake.

## 5. Conclusions

New Zealand blackcurrant extract seems to be beneficial in repeated short-distance cycling time trials for overall performance. The ergogenic effect of New Zealand blackcurrant may be due to blood flow and anti-oxidant effects on fatigue mechanisms of high-intensity exercise.

## Figures and Tables

**Table 1:** Subject characteristics of male cyclists.

Age (years)	Height (cm)	Body Mass (kg)	$\dot{V}$O_2max_ (mL·kg^−1^·min^−1^)	HR_max_ (beats·min^−1^)	Body Fat (%)
30 ± 12	179 ± 7	74 ± 9	55 ± 7	189 ± 14	11 ± 3

$\dot{V}$O_2max_, maximum oxygen uptake, HR_max_, maximal heart rate. Values are means ± SD.
